# Medical and Surgical Uses of Electricity

**Published:** 1879-02

**Authors:** 


*A Practical Treatise on the Medical and Surgical Uses of Electricity,* including Local and General Faradizations; Local and Central Galvanization; Electrolysis and Galvano-Cautery. By GEORGE M. BEARD, A. M., M. D., and A. D. ROCKWELL, A. M., M. D. Second edition *Revised, Enlarged* and mostly Re-written, with nearly two hundred Illustrations. New York: William Wood & Co., 27 Great Jones street, 1878.

This work was first published in 1871, and is well known to the profession as a standard upon electricity.

The science of electro-therapeutics has progressed both with the profession and public very rapidly for the past few years, though it still remains largely in the hands of itinerant manipulators, who advertise and use it indiscriminately and, for the most part, ignorantly. This book is a comprehensive, well written text book upon the subject, and our readers may depend upon its containing all that is known, with full directions for the use of electricity by those desiring to make a trial of its effects.

